# Supplementary material for: Are carbon emissions trading and green financial instruments synergistic? -Comprehensive quantitative research based on content analysis
Source: PLoS One. 2024 Mar 7;19(3):e0298601. doi: 10.1371/journal.pone.0298601 (PMC10919601; doi:10.1371/journal.pone.0298601)
Supplement: S1 Data — (ZIP) [file pone.0298601.s001.zip › Data/地级市Z_F工作报告.zip!/║■─╧/┬a╡╫╩╨/└┤╫╘╣1⁄2╓┌║┼í╛┬φ┐╦╩2╛▌═°í┐_2011.docx]

各位代表：

　　现在，我代表市人民政府，向大会作工作报告，请连同《娄底市国民经济和社会发展第十二个五年规划纲要（草案）》一并予以审查，并请各位政协委员和其他列席人员提出意见。

一、2010年和“十一五”工作回顾

　　2010年，是经济形势较为复杂的一年，也是全市上下积极应对、奋发努力、取得显著成绩的一年。市政府在市委的坚强领导下，在市人大、市政协的监督和大力支持下，深入实践科学发展观，贯彻落实中央、省里和市委的决策部署，坚定实施“科学发展、加速赶超”战略，确保了全市经济社会平稳较快发展，圆满完成了市三届人大二次会议确定的各项目标任务。

　　全年实现生产总值678亿元，增长14.7%（预计数，下同）；完成财政总收入56.3亿元，增长20.96%，其中一般预算收入30.01亿元，增长24.83%；城镇居民人均可支配收入15086元，农民人均纯收入3364元，分别增长12.4%、8.6%；人口自然增长率6.16‰；城镇登记失业率4.5%；万元GDP能耗、二氧化硫排放量和化学需氧量等控制指标完成省定任务；亿元GDP生产安全事故死亡率下降43.26%。各项指标均圆满完成年初确定的目标任务。

　　一年来，我们主要抓了以下五个方面的工作：

　　（一）着力做强产业，确保发展速度

　　工业快速增长。扎实开展“工业企业服务年”活动，实现工业增加值331亿元，增长19.8%。规模工业企业达到664户，总产值首次突破千亿元大关，实现增加值302亿元，增长21.9%。传统产业改造步伐加快，完成技改投资160亿元，增长16.8%。大丰和绿色动力科技园、金华特种车辆、荣诚二期成品鞋生产线等新兴产业项目开工建设。继续开展“科技创新年”活动，完成全社会研发投入5.8亿元，申请专利980件，授权423件，分别增长6.5%、52 %，获省科技进步奖4项，科技示范基地达到132个，高新技术企业达到54家，完成高新技术产品增加值104亿元，增长54.5%。

　　农业基础稳固。实现农业增加值98.8 亿元，增长5.1%。粮食播种面积406.05万亩，总产163.3万吨。农业产业化水平提升，湖南黑猪、湘中黑牛等特色产业不断壮大，种养大户超过5万户，市级以上龙头企业达到102家，农产品加工产业完成销售收入59.41亿元，增长24.3%。农民专业合作组织达到459家，流转土地36.32万亩，带动农户17.53万户。休闲农业企业达到252家。农产品质量安全监管体系不断完善，获得无公害农产品质量认证87个、绿色食品认证25个、有机食品认证7个。科普惠农跃上新台阶。农业机械化率达到34%。新化县被评为全国粮食生产先进县，双峰县获得全省粮食生产标兵县和“中国碾米机之乡”称号，涟源市被评为全省农业产业化发展先进市。蔬菜示范基地建设卓有成效，娄底城区万亩蔬菜基地初步建成。

　　三产活力增强。实现第三产业增加值214.2亿元，增长12%。完成社会消费品零售总额219.6亿元，增长18.3%。进出口总额突破15亿美元，增长76.5%。在全省率先启动乡村旅游富民工程，成功创建中国优秀旅游城市，旅游接待750万人次、总收入39亿元，分别增长38%、42%。继续推进“汽车、家电以旧换新”、“家电、汽摩下乡”、“新网工程”和“万村千乡市场工程”，发放财政补贴1.99亿元，带动直接消费18亿元。房地产、交通运输、邮电通信、批发零售等传统服务业不断壮大，金融、保险、“网上供销社”等现代服务业加快发展。

　（二）着力扩大投资，增强发展后劲

　　加快项目建设。继续开展“项目建设年”活动，全年施工项目909个，新开工项目646个，分别增长27.5%、23.0%。完成全社会固定资产投资400亿元，增长31.28%。

大力招商引资。积极参加“世博会”、“厦交会”等招商活动，成功举办首届“娄商大会”，承办“湖南省第二届农业机械、矿山机械、电子陶瓷博览会”。重型轧辊、柳工再制造等产业项目签约落户我市。实现内联引资110.4亿元，实际利用外资1.62亿美元，分别增长37.1%、20%。

扩大金融信贷。优化金融环境，开展金融扶持中小企业800家工程，招商银行、华融湘江银行筹备设立营业机构，村镇银行、农村商业银行筹备工作扎实推进，新增汇丰和源方2家小额贷款公司。9家企业进入省重点上市后备企业资源库，5家企业得到省财政上市专项引导资金扶持。各类金融机构贷款余额439亿元，净增101.1亿元。

　　（三）着力夯实基础，巩固发展平台

　　推进交通建设。沪昆铁路客运专线和洛湛铁路娄邵线改造全面施工；娄新、安邵、新溆3条高速公路加快建设，新开工娄衡、娄益、娄长3条高速公路；7条干线公路改造完成投资10.05亿元；农村公路完成通畅工程900公里，改造危桥19座，完成投资6亿元。

　　推进城镇建设。启动娄底城市总体规划新一轮修编，完成市政公用基础设施投资25.2亿元，城镇化率达到37.5%。潭邵高速娄底连接线扩建、甘桂路、涟水与孙水风光带等重点工程加快建设。全市6个污水处理厂建成投入使用，管网基本配套。县市城区生活垃圾卫生填埋场建成运行。市本级创建国家园林城市、国家卫生城市、全国文明城市工作扎实推进，县市创建工作积极开展，双峰县成功创建省级卫生城市，全市违法建设专项整治成效明显。小城镇加快发展，涌现了一批全国全省重点镇和示范镇。

　　推进园区建设。园区集聚功能进一步彰显，园区工业增加值占全市工业增加值的比重达到28.7%。娄底经济开发区获批国家新型工业化产业示范基地，被评为全省承接产业转移示范园区和最具产业影响力园区。水府“两型”示范区规划编制全面完成，万宝新区和东部新区启动建设。

　　推进生态建设。圆满完成节能减排任务，关闭淘汰顺鑫钢铁等落后产能项目52个，否决不符合国家产业政策和环保要求的项目65个。14家企业参加全国千家和全省百家企业节能行动成效明显。加强环境治理，整顿规范矿产资源开发秩序，锡矿山地区被列入湘江流域重金属污染治理重点项目，并启动实施。大力实施“三边”造林和“四旁”绿化，义务植树889万株，造林17万亩，森林覆盖率达到48.13%，获全省林业建设目标管理考核“湘林杯”。加强用地保障，重点工程、民生工程与基础设施建设用地占审批总量的40%，连续11年实现耕地总量占补动态平衡。

　　推进新农村建设。新建沼气池5026口，解决25.9万农村人口饮水安全问题，治理病险水库18座，完成水利建设投资4.2亿元，我市再次夺取全省水利建设“芙蓉杯”。清洁工程及村庄环境整治有序推进，村容村貌得到较大改观。冷水江市被评为全省新农村建设先进单位。投入扶贫资金2.8亿元，减少贫困人口3.2万人。争取移民后扶资金2.3亿元，受益移民32万人，新化县获全国水库移民后扶政策实施工作先进集体。革命老区、农民负担监管、“三冬”生产等工作扎实开展，森林防火、农林病虫害防治、气象水文信息和技术服务、农资供应和价格调控、动物疫病防控等工作进一步加强。娄星区被评为全省动物防疫工作先进单位。

（四）着力改善民生，凝聚发展合力

　　战胜4次特大暴雨洪灾，最大限度保障了人民群众生命财产安全和灾后生产生活。市防汛抗旱指挥部被评为全国防汛抗旱工作先进集体。

　　倾力为民办实事，圆满完成28项实事任务。财政对农业、社保、医疗卫生、教育、环保等民生支出达到60.8亿元，占财政支出的57.7%。新增廉租住房8127套，新建经济适用房500套，改造农村危房3107户，灾后重建1580户，发放住房公积金贷款6.8亿元。娄星区获全省为民办实事农村清洁工程先进单位。

　　大力促进就业创业，培训10.81万人，城镇新增就业和安置再就业3.15万人，下岗失业人员实现再就业1.74万人，农村劳动力转移就业86.53万人，稳定和安置残疾人就业8345人。

　　养老、失业、医疗、工伤、生育和新型农村社会养老保险覆盖面不断扩大，272.57万人次受益。城市低保应保尽保，农村低保提质扩面，发放低保金2.44亿元，新建和改扩建敬老院8所，五保对象集中供养标准年人均达到4000元以上，保障水平普遍提高。筹措慈善首创基金1600万元，慈善总会正式成立。医疗救助、阳光行动卓有成效。冷水江市被评为全国残疾人社区康复示范市。

　　教育事业加快发展，有效控制城区中小学校入学新生班额过大问题，高中招生制度改革圆满完成，教学质量稳步提升，高考录取率达到78.68%，提高15.15%。建设农村义务教育合格学校58所，维修改造农村中小学校舍158所。娄底职业技术学院成功申报国家级重点职业院校，娄底电大新址建设与娄底技师学院筹建有效推进，娄底卫校升专工作进展顺利。双峰县被评为全省建设教育强县工作先进县和省义务教育均衡发展先进县。

　　大型花鼓戏《花落花开》被中组部列入全国农村党员远程教育教材，电影《爱上油菜花》列为国务院农民工电影周活动展映电影，新建39个乡镇综合文化站，建成851家农家书屋。城区数字电视整体平移，有线电视双向网改全面启动，新闻出版、广播电视基础设施进一步完善，传媒影响力进一步提升。市体育中心建设即将完工，成功申办省第十二届运动会。

　　医疗卫生体制改革进一步深化，基本药物制度试点稳步推进，公共卫生、农村卫生、社区卫生均衡发展，卫生监督、基础免疫工作扎实有效，重大传染病有效控制，免费完成农村氟中毒病区改炉改灶3.1万口，9类国家基本公共卫生服务项目全面实施。人口与计生工作连续四年保持全省先进，我市被确定为“全国青少年健康人格教育工程试点单位”。

（五）着力依法行政，强化发展保障

　　主动接受人大法律监督、工作监督和政协民主监督，认真办理人大议案1件、代表建议221件、人大常委会和主任会议审议意见18件、政协建议1件、委员提案198件。严格执行《湖南省行政程序规定》，加强规范性文件管理与行政复议、行政应诉工作，在全省首创规范性文件管理人和听证主持人制度。圆满完成“五五”普法任务。

　　集中开展“创先争优”和“机关行政效能建设年”活动，进一步转变政府职能，简化行政审批环节，规范权力运行制度，建设项目审批提速50%以上。加强对财政预算执行和干部任期经济责任审计，审计执行力提高。大力优化经济环境，在全省率先实行领导挂点、部门包干优化工作责任制，对县市区经济环境实行指数考评，集中开展整治“三强”、严肃责任追究专项行动，查处典型案件48起，对75名党员干部严肃问责。

　　实施优秀人才引进专项编制制度，引进硕士学位以上优秀专业人才134人，公选副处以上干部13人。政府机构改革基本完成，林权制度改革完成主体改革任务，“省直管县”财政体制改革、国企改革改制扫尾、人口计生综合改革等各项改革统筹推进。

　　强化安全生产责任落实，安全生产形势持续好转，事故总死亡人数下降28.13%，煤矿等重点高危行业事故明显减少，杜绝了重大事故，我市在全省考核中排名第一，被评为全省先进。食品药品安全专项整治效果明显。加强信访维稳源头防控，赴省进京上访总量持续下降，实现全国“两会”等特护期赴省进京非正常上访“零登记”目标，信访工作获全省先进，集中治理信访积案经验在全国推广。全市社会政治大局持续稳定，社会治安综合治理工作在全省排名上升，公众对“社会治安状况变化趋势”评价排名全省第一。

　　军队和国防后备力量建设稳步推进，公安、消防、武警队伍建设不断加强，在全市经济社会发展特别是抗灾救灾和维护社会稳定中做出了重要贡献。涟源市荣获全省双拥模范城市称号。统计服务质量明显提高，第六次人口普查进展顺利。第二轮修志全面启动。国资监管、民族、宗教、人防、对台、外事侨务、档案、仲裁、保密、地震和工会、共青团、妇联等各项工作取得长足进步。

　　各位代表，丰收的2010年，为“十一五”划上了一个圆满句号。经过五年奋斗，我们全面完成了“十一五”规划确定的各项目标任务，开创了“科学发展、加速赶超”的崭新局面。

这五年，是我市科学发展加速赶超、综合实力大幅跃升的五年。面对历史罕见的低温雨雪冰冻灾害，面对影响空前的国际金融危机，面对突如其来的特大洪涝灾害，市委、市政府审时度势，果断决策，科学应对，带领全市人民以超常规举措，确保经济超常规发展。生产总值2010年是2005年的2.24倍，年均增长13.1%，高出“十五”时期3.1个百分点。人均生产总值2010年是2005年的2.1倍，达到1.7万元。全社会固定资产投资2010年是2005年的3.4倍，年均增长27.7%。财政一般预算收入2010年是2005年的2.45倍，年均增长19.58%。我市作为环长株潭城市群重要一极，在全省的综合竞争力明显提升。

　　这五年，是我市产业结构加快调整、发展后劲显著增强的五年。以存量引增量，加快国企改革攻坚，改制重组企业重现生机与活力。以技改促升级，五年淘汰落后产能项目192个，“五小”企业基本关停到位，传统产业优势更加巩固。以创新增活力，大力实施建设创新娄底战略，在全国首创了农村科技合作社，在全省率先建立了产业技术创新战略联盟，获批全国知识产权试点城市和国家高新技术产业基地。以开放促发展，五年累计内联引资350亿元，实际利用外资5.99亿美元，引进了一批符合国家产业政策、符合新型工业化要求的大项目、好项目，尤其是汽车板电工钢、三一中源新材料工业园等战略性产业项目纷纷落户并开工建设。第三产业加速发展，旅游业不断壮大，制鞋、服饰、现代物流等产业蓬勃发展。三次产业结构从2005年的16.6:47.4:36调整为14.6:53.8:31.6。

　　这五年，是我市基础建设突飞猛进、城乡面貌日新月异的五年。交通建设实现大跨越，1条高铁新建、1条铁路改造、6条高速公路全面开工，打响了7条干线公路改造三年大会战，公路总里程达到1.69万公里，娄底机场进入预可研，我市东南西北四个出口即将全面打通，“十一五”是我市交通建设投入最大、速度最快、成效最显著的时期。城镇建设实现大提质，市、县（市）城区和中小城镇骨架不断拓展，功能不断完善，品位不断提升，娄底中心城区建成区面积达到42平方公里，城市人口达到39万，我市先后荣获省级卫生城市、省级文明城市、全国绿化模范城市、中国优秀旅游城市称号，城市美誉度和知名度空前提高。新农村建设实现大发展，公路村村通、广播电视村村通、互联网络乡乡通全面实现，农田水利基础设施建设、防汛抗旱和供水、供电、供气能力显著增强。基础的不断夯实为经济社会快速发展插上了腾飞的翅膀。

　　这五年，是我市社会民生持续改善、人民群众受惠最多的五年。践行政府承诺，连续五年为民办实事，倾力解决了住房保障、安全饮水、就业再就业保障等一批关系群众切身利益的问题。有效落实强农惠农政策，全面推广新型农村合作医疗制度，不断拓宽社会保障覆盖范围，让广大人民群众共享改革发展成果。城镇居民人均可支配收入2010年是2005年的1.89倍，年均增长13.5%，农民人均纯收入2010年是2005年的1.43倍，年均增长7.5%；五年内减少低收入人口13.1万。加强资水、涟水、孙水和采煤、采矿沉陷区综合治理，加大污水处理厂和垃圾填埋场建设投入，推进农村环境整治，全面加快生态建设。加强安全生产工作，推进安全生产示范乡镇建设，年度事故总死亡人数年均下降15%，从事故重灾区转变为全省安全生产先进市州。加强社会治安综合治理，协调发展教育、卫生、文化、体育、人口与计生等各项社会事业，加快构建和谐社会，人民群众的精神文化生活更加丰富，生活质量明显提高。

　　这五年，是我市齐心协力团结奋进、发展活力全面迸发的五年。面对改革发展的新情况、新问题，全市上下不犹豫、不折腾、不懈怠，心往一处想，劲往一处使，因势利导，顺时而动，抢抓机遇，化“危”为“机”，战胜了一次又一次挑战与困难，描绘了一幅“科学发展、加速赶超”的路线图，谱写了一部负重前行、催人奋进的赶超史，唱响了一首励精图治、众志成城的创业歌。五年的奋斗历程，五年的辉煌成就，统一了思想，凝聚了人心，激励了斗志，焕发了活力，在全市上下营造了心齐气顺劲足的发展氛围。

　　回顾五年历程，我们深切体会到，这些成绩的取得，得益于市委的坚强领导，得益于市人大、市政协的监督支持，得益于历届领导班子打下的坚实基础，得益于各级各部门和社会各界的团结合作，得益于全市人民的共同努力。在此，我代表市人民政府，向全市人民，向各位代表和委员，向各民主党派、工商联、无党派人士、各人民团体和社会各界人士，向驻娄人民解放军、武警和消防官兵、民兵预备役人员、公安干警，向中央、省属驻娄单位和企业的同志，向各位离退休老领导、老同志，以及所有关心、支持我市建设和发展的朋友们，表示衷心的感谢并致以崇高的敬意！

　　五年的不懈努力，创造了改革发展的丰硕成果；五年的生动实践，也积累了许多宝贵经验：一是必须始终深入贯彻落实科学发展观，解放思想，实事求是，与时俱进，不断探索切合我市实际的发展新路子；二是必须始终坚持科学研判形势，审时度势，顺势而为，努力抢抓发展机遇；三是必须始终坚持改革开放，着力构建有利于科学发展、加快发展的体制机制，增强发展活力；四是必须始终坚持改善民生，维护社会和谐稳定，用发展的成果造福人民；五是必须始终坚持加强政府自身建设，大力推进政府管理创新，提高领导科学发展的水平和驾驭复杂局面的能力。

　　在肯定成绩的同时，我们也清醒地看到，我市发展中还存在很多困难和问题，主要是：综合实力不强，加速赶超压力大；产业结构不合理，资源型高能耗的产业比重较大，加快转变发展方式的任务还很艰巨；县市区发展不平衡，农民持续增收难度大，加快城乡、区域统筹还需花大力气；低收入群体生活困难，一些事关群众切身利益的难点问题亟待解决，民生保障任务很重；干部作风、办事效率有待提高，发展环境有待进一步优化。所有这些，都需要我们高度重视，并在今后工作中采取有力措施加以解决。

二、“十二五”发展思路

　　“十二五”时期，既是我市的重要战略机遇期，也是发展关键期，更是加快转变经济发展方式、全面推进“两型社会”建设的攻坚期。从国际环境看，当今世界正处于大发展、大变革、大调整的重要时期，后金融危机时期国际经济环境更加复杂，科技竞争更加激烈；从国内环境看，国家推动经济发展方式转变和结构调整力度加大，促进中部崛起和支持资源型城市转型等政策力度也在加大；我省“四化两型”战略全面实施，环长株潭城市群建设提速。总的来看，未来我市发展的宏观环境既有挑战，更有机遇，机遇大于挑战。

　　“十二五”时期的指导思想是：以邓小平理论和“三个代表”重要思想为指导，深入贯彻落实科学发展观，贯彻落实党的十七届五中全会、省委九届十次全会、市委三届七次全会精神，大力实施“科学发展、加速赶超”战略，以加快转变经济发展方式为主线，以对接融入长株潭为路径，突出“两型”引领、结构调整、创新驱动、项目支撑、改革开放、民生改善，加快推进新型工业化、农业现代化、新型城镇化和信息化，努力建设活力娄底、魅力娄底、富裕娄底、和谐娄底，为全面建设小康社会奠定坚实基础。

　　“十二五”期间的奋斗目标是：生产总值年均增长12.5%以上，到2015年突破1300亿元；财政一般预算收入年均增长13%以上；全社会固定资产投资五年累计完成4200亿元，年均增长25%以上；全社会消费品零售总额年均增长18%以上；城镇登记失业率控制在4.5%以内；城镇居民人均可支配收入年均增长11%以上，农民人均纯收入年均增长11.5%以上；人口自然增长率控制在7.5‰以内；亿元GDP生产安全事故死亡率等相对控制指标达到全省先进水平；节能减排完成省定目标。初步建成“三基地一中心一枢纽”，努力打造新型工业城市、交通枢纽城市、生态宜居城市、和谐文明城市。

　　主要任务是做到“六个坚持”：

　　（一）坚持“四化”带动，构建现代产业体系。着眼做大规模、调优结构，推进新型工业化，加快园区建设，延伸产业链条，促进产业集聚，实施“153211”工程，即打造精品钢材及薄板深加工1000亿产业集群、煤炭及深加工500亿产业集群、汽车及机械制造300亿产业集群、有色金属及深加工200亿产业集群、建材与农产品深加工2个100亿产业集群。着眼提高农业比较效益、促进农民持续增收，推进农业现代化，稳定粮食生产，大力发展精深加工，培育壮大一批龙头企业，打造长株潭重要农产品供应基地。着眼集聚人口、承载产业，推进新型城镇化，加快产业填充，提升生活性服务业，发展生产性服务业。着眼“四化”融合、培育新的经济增长点，推进信息化，加快信息技术、信息资源开发和应用，发展信息产业，建设数字娄底。力争“十二五”期末，三次产业结构调整为9:53:38，形成新型工业、现代农业和现代服务业互相融合、协调发展、富有竞争力的现代产业体系，全面完成我市产业升级换代。

（二）坚持“两型”引领，打造绿色娄底。注重资源节约，倡导绿色消费，积极创建国家级循环经济试点城市。注重环境保护，加强污染治理，着力防治工业源头、城市区域、重点流域和农村面源污染，城镇污水处理率和生活垃圾无害化处理率分别达到80%、100%。注重生态建设，加强林地、湿地、风景名胜区及生态脆弱地区的保护和修复，统筹推进城乡绿化，森林覆盖率达到50%。注重示范带动，加快水府示范片区建设，着力打造生态优良、系统完整、功能完备、舒适怡人的山水生态新区。

　　（三）坚持基础先行，强化发展保障。立足对接融入长株潭，着力完善城镇路网和基础设施配套，壮大“一心两翼”城镇带，打造环长株潭重要节点城市，城镇化率达到46%，娄底中心城区建成区面积达到52平方公里以上。立足夯实农业基础，加强城乡防洪、河道整治、农村水利、乡村公路、农村电网改造等工程建设，提高农业综合生产能力，推进新农村建设。立足构建大交通网络，加快建设“六纵八横”主干公路网，建成沪昆铁路客运专线（娄底段），完成洛湛铁路娄邵线、娄益线改造，争取开工建设安张娄衡铁路、娄底机场，加强港口、码头和油气管网设施建设，形成以铁路、公路、水运、机场、城市交通为支撑的综合交通枢纽。经过五年努力，彻底改写“内无好路、外无出路”的历史，打造市域和融入长株潭“一小时经济圈”，正式进入高铁和高速发展时代。

　　（四）坚持科教兴市和人才强市，建设创新娄底。优先发展教育，整合教育资源，加大教育投入，均衡发展基础教育，加快发展学前教育和民办教育，大力发展职业教育和成人教育，稳步发展高等教育，建设教育强市。加快自主创新，全社会研发投入占生产总值比率达到2%以上，建设省级工程技术中心5个以上，60%的规模以上工业企业建立研发中心，初步建成区域创新体系。高度重视人才，加大人才培养和引进力度，建立健全人才评价、选拔、任用和激励机制，着力建设党政领导人才、企业经营管理人才、专业技术人才、高技能人才和农村实用人才五支队伍，为经济社会发展提供智力支撑。

　　（五）坚持改革开放，增强发展活力。加强经济协作与对外联络，大力引进战略投资者，加快发展外向型经济，力争“十二五”实际利用外资14亿美元以上，内联引资800亿元，2015年进出口总额突破20亿美元。推进重点领域和关键环节改革，加快土地、资本、劳动力、技术等要素市场建设，设立中小企业创业投资引导资金，逐步构建中小企业社会化投融资体系，基本形成符合“两型社会”建设要求的体制机制和发展模式。

　　（六）坚持统筹兼顾，切实改善民生。加快统筹城乡发展，促进规划、产业布局、基础设施建设和公共服务向农村延伸，进一步扩大社会保障覆盖面，实现新型农村社会养老保险全覆盖。把就业摆在更加突出的位置，全面提高劳动者就业创业能力，多渠道增加就业岗位。发展社会福利和慈善事业，健全社会救助体系，全面推进库区和扶贫建设，有效解决中低收入群体的实际困难。深入实施文化强市战略，全面提升文化软实力。加强医疗卫生、体育、广播电影电视、人口计生、住房保障工作。办好湖南省第十二届运动会和第九届残疾人运动会。加强社会建设和管理，推进综治维稳和信访工作，加强安全生产，促进社会和谐稳定。

三、2011年工作重点

　　2011年是“十二五”开局之年，是中国共产党建党九十周年，做好今年工作意义非常重大。我们必须认真贯彻落实中央、省委和市委经济工作会议精神，坚定实施“科学发展、加速赶超”战略，突出“工业主导、基础先行、项目带动、协调发展”四个重点，继续开展“工业企业服务年”、“项目建设年”和“科技创新年”活动，全面推进“四化两型”建设，为“十二五”规划的顺利实施开好局、起好步。

　　今年全市经济社会发展的主要预期目标是：生产总值增长12%以上；规模以上工业增加值增长16%以上；财政一般预算收入增长13%以上；全社会固定资产投资增长20%以上；进出口总额增长10%以上；城镇居民人均可支配收入和农民人均纯收入均增长11%以上；人口自然增长率控制在7.3‰以内；节能减排完成省定目标。为实现上述目标，今年将着重抓好以下八个方面的工作：

　　（一）加快推进新型工业化

　　改造提升传统产业。以高端化、高新化、“两型”化、规模化为目标，推动冶金、能源、建材、机械、化工等传统优势产业提质升级。壮大涟钢、冷钢等骨干企业，推进和实施方瑞钢管、三星锻造、华新水泥、汇源新材料、双峰不锈钢产业园、湖南海螺二期等一批技术改造项目，完成技改投资150亿元。加快煤矿集团化、机械化、标准化建设。

　　大力发展战略性新兴产业。深入实施战略性新兴产业发展规划，强化政策支持，选择有一定基础、具有比较优势的领域先行发展，促进规模扩张，加快集群集聚。重点发展先进装备制造、新材料、新能源及电动汽车、电子信息等四大产业，鼓励和支持发展生物、节能环保、文化创意等新兴产业，加快大丰和绿色动力科技园、金华车辆等项目建设，着力打造汽车制造产业集群。

　　加快发展园区工业。加快园区基础设施和标准厂房建设，促进产业向园区集聚，强化园区综合管理职能和管理职权，赋予园区更多自主权。鼓励支持园区走“特色化”、“差异化”、“专业化”路子，支持娄底经济开发区创建国家级经济技术开发区。加快娄底铁路口岸和海关建设，有效承接产业转移。确保园区规模工业增加值占全市规模工业增加值的比重提高1至2个百分点。

切实提高自主创新能力。深入推进产学研结合，巩固拓展产业技术创新战略联盟，实施“质量兴企、品牌立市”战略。建设闪星锑业理化分析实验室、红太阳电池材料工程研究中心、轻型低速电动汽车及关键零部件等研发中心。打造娄底市薄板材料新型装备制造产业基地服务平台和新化特种陶瓷、涟源矿山机械、双峰农业机械公共技术与产业化服务平台。确保高新技术产业和战略性新兴产业产品增加值占规模工业增加值的比重达到25%以上。

　　（二）加快推进农业现代化

　　毫不放松抓好农业生产。严格保护耕地，扩大优质稻种植面积，确保粮食总产165万吨以上。下大力气抓好“菜篮子”工程，高标准建设好杉山、小碧、桥头河、中阳、青树坪等蔬菜示范基地，确保蔬菜种植面积增加1.5万亩，湖南黑猪和湘中黑牛产业产值增长10%以上，生猪、肉牛出栏分别增长4%、8%以上，保障主要农产品有效供给。实施优质种苗工程。大力发展特色农业，新造油茶林8.3万亩，低改油茶林2.7万亩，低改南竹30万亩，发展油菜50万亩、软籽石榴等名特优水果2万亩。

　　努力改善农业发展基础条件。加大水利建设投入，突出抓好小型病险水库治理、农村饮水安全、大中型灌区续建配套、小型农田水利设施、城市防洪、农村电气化等重点工程，强化水资源管理，做好第一次全国水利普查工作。加快农村公路建设，争取完成通畅工程400公里。推广高效节能农机具，大力推进农业机械化。完成农业科技培训11万人次、职业技能培训3万人次。加快防汛抗旱指挥中心和物资储备中心建设。加强农产品质量安全监管、气象水文预报、山洪灾害预警、森林防火、动物防疫、农林有害生物防控等体系建设，提高综合防控能力。

　　积极推进农业产业化经营。延伸农产品加工产业链，力争农产品深加工产值增长30%以上。积极引进培育龙头企业，力争国家级农业产业化龙头企业实现零的突破。加快土地依法有序流转，大力发展农村专业合作组织，促进农业向规模化、集约化、产业化、品牌化发展。发展“网上供销社”，利用信息手段拓展农产品市场。

（三）加快推进新型城镇化

　　更大力度推进中心城区提质扩容。完成娄底城市总体规划新一轮修编，重点抓好甘桂路、西气东输二线、湘潭至娄底成品油管输工程等项目建设，继续推进中心城区河道治理及流域管理。深入推进“三个创建”工作，抓好株山公园、仙女寨植物园、潭邵高速娄底连接线绿色通道等重点绿化工程项目，推进街道、公园、广场、院落的增绿提质，确保实现国家园林城市创建目标。广泛开展爱国卫生运动，加强城市管理综合执法，继续大力整治违法建筑，加强社区环境卫生管理，基本完成创建国家卫生城市主要工作任务。推进社区建设，加强市民文明教育，培育健康向上的文明风尚，争创“全国创建文明城市工作先进城市”。

　　更高标准加快县市城区与小城镇建设。冷水江市加快推进城市东扩，完成资江大道、平安大道、群丰大道建设，扎实推进创建省级卫生城市。涟源市积极实施北扩东延战略，加快城东新区和市民服务中心建设，重点建设石马路、梅城路、岛石路、人民东路等基础设施，创建省级卫生城市和省级文明城市。双峰县加快第二水源工程建设及水厂提质改造，推进艺芳新城、城区景观改造、城市堤防及湄水河两岸风光带建设。新化县加强县城安全供水、北塔区综合改造、资江三桥、城东防洪堤建设，创建省级卫生城市和省级文明城市。以中心镇和示范镇为重点，增强小城镇对人口和产业的吸纳和承载能力。全市城镇化率提高2个百分点。

积极推进城乡统筹发展。以冷水江市城乡统筹、整体推进新农村建设试点为先导，全面完成各县市区土地利用总体规划修编、县域村庄布点规划。加强城乡之间规划、基础设施、公共服务、基本保障的对接，促进城乡产业融合，努力缩小城乡差距。大力发展县域经济，统筹项目布局和政策扶持，促进各县市区平衡发展，鼓励和支持有条件的地区率先发展。

　　（四）加快推进信息化

加强信息基础设施建设。以统一城市通信管道资源建设为突破口，完善、整合网络资源，促进电信网、广电网、互联网“三网融合”。推进城市骨干信息管网向农村辐射延伸，扩大网络覆盖面。加强电子政务建设与信息资源开发，促进部门信息共享，提供透明、方便、快捷的网上政务服务。

大力发展信息产业。推进娄底太和信息产业园等基地建设，积极发展电子信息材料、电子元器件等信息产品制造业，把信息产业培育成新的经济增长点。推进电信业由传统运营业向信息服务业转变，培育互联网增值服务。以全国移动商务在湖南开展试点为契机，着力发展电子商务、物联网、虚拟经营、连锁经营等信息服务业。

　　（五）加快推进“两型社会”建设

推进示范区建设。加快万宝新区和东部新区建设，加快娄星北部经济园区顶层设计。完成潭邵高速娄底连接线扩建工程，加快娄星南路、仙女大道、高丰路、高铁南站站前广场、娄益高速连接线、园区内部道路、22万伏变电站等基础设施建设，加快鸿帆铝业、湖南煤机等已落地项目的建设进度，推进红宇耐磨、新世界建材城、移动大厦等项目动工建设，加快引进战略性新兴产业和绿色环保产业项目，确保年内完成投资20亿元，构建新区基本轮廓。

狠抓节能减排。增强节能意识，扎实推进企业节能行动，发展循环经济，大力促进社会节能，实施瓦斯抽采利用等项目。坚持源头治理，严格落实环保“三同时”制度，坚决杜绝高能耗、高污染产业与项目进驻。加快市中心城区一污水处理厂二期工程建设，实施湖南宜化生产污水综合治理工程，完善已建污水处理厂配套设施，提高污水集中处理率。严格责任目标考核，进一步形成节能减排协作机制、监督机制和约束机制。

加强环境治理。启动锡矿山地区砷碱渣污染治理项目，推进涟钢和冷钢周边环境治理。保护好白马、大江口、双江、车田江等水库供水水源，推进资水、涟水、孙水等流域综合治理。支持有条件的乡镇建设污水和垃圾处理设施。全面实施农村环境卫生专项整治三年行动计划。强化重点行业和企业强制性清洁生产审核。加快造林绿化，森林覆盖率达到48.63%。

　　（六）加快发展服务业

　　做大做强生活性服务业。加快区域性商业中心和特色商业街建设，培育娄底汽贸城等区域性专业市场，逐步推进农贸市场标准化改造，提升餐饮、住宿、商贸等传统服务业水平。加快发展农业生产资料现代经营服务网络，促进“农超对接”。加大商务综合执法力度，完善供需和物价调控体系，继续推进“万村千乡市场工程”、“双百市场工程”、“新网工程”等活动，拉动直接消费。确保社会消费品零售总额增长18%。

大力发展生产性服务业。加快娄底湘中国际（陆港）物流园等项目建设，发展现代物流业。鼓励发展金融、信息、保险、证券等生产性服务业。实施“科技兴贸”战略，加快发展加工贸易和服务外包产业，引导鼓励民营企业开展对外贸易，加快农产品省级出口基地创建，建好娄底出口产品原产地签证处。全年实现进出口总额增长10%以上，加工贸易增长15%以上。

　　进一步壮大文化和旅游业。加快构建以文化旅游、现代传媒、新闻出版、广告会展等联动发展的产业框架。全面实施乡村旅游富民工程，推进旅游与农业、林业、水利、新型城镇化融合发展。抓好水府庙国家湿地公园旅游开发，打造国际影视新城。建设以新化为核心的湖南大梅山文化旅游经济协作区。推进紫鹊界梯田、中华蚩尤始祖文化园、曾国藩故里、梅山龙宫、大熊山、龙山、湄江、波月洞等重点景区的提质升级。扩大中国优秀旅游城市品牌影响力，把旅游业培育成为我市战略性支柱产业，确保旅游接待人数和总收入均增长30%以上。

　（七）加快推进项目建设

　　抓好重点项目。交通项目完成投资120亿元,力争140亿元。重点抓好2条铁路、6条高速公路、7条干线公路和娄长高速连接线、新星南路等项目建设。加快张桂高速（娄底段）、双衡高速、娄长高速经涟源至新化琅塘高速公路段、沪昆铁路客运专线娄底南站至双峰县城城际连接线的前期工作，继续推进冷水江东站扩能改造。全面竣工“十一五”干线公路结转项目，争取冷水江沙塘湾至禾青公路、涟源大塘至雷鸣洞、双峰香铺坳至杏子铺、新化吉庆经温塘至田坪等干线公路改造项目开工。工业项目完成投资180亿元，力争200亿元。重点抓好汽车板电工钢、中兴液压、三一中源新材料工业园、华润雪花啤酒、重型轧辊、金华车辆、文昌科技园、五江循环经济工业园、兴昂荣诚晟丰1200万双成品鞋生产线、3万吨乙撑胺、天宝紧固件二期、映鸿新材料工业园等项目建设。农林水项目完成投资15亿元，重点抓好娄底中心城区城市防洪堤、长防林、退耕还林等生态环境建设、新增粮食产能工程、移民后期扶持项目、农牧渔服务业等项目建设。社会事业项目完成投资11亿元，重点抓好市卫生学校升专扩建、市卫生监督所新建、市体育公园、市会展中心、市委接待处改造、市委党校综合培训楼、市环境监控中心、娄底技师学院和县市区人民医院改扩建等项目。城市基础设施完成投资28亿元，房地产开发完成投资41亿元，城镇以下固定资产完成投资90亿元。

　　强化资金保障。大力招商引资，突出产业招商、以商引商，注重发挥商会组织与娄商作用，重点加强与世界、全国500强、央企和沿海产业的对接合作，高起点承接产业、资金、技术转移，实现内联引资130亿元以上，力争150亿元，到位外资1.8亿美元。加强各类融资，培育和引进金融机构，争取年内引进1至2家股份制银行设立分行，争取筹建1家村镇银行和5家小额贷款公司，力争全市新增贷款比年初增加20%以上。支持成长性好的中小企业尽快上市。鼓励民间投资，大力引导民间资本参与战略性新兴产业和城镇基础设施等项目建设。

　　加强财政管理。顺应“省直管县”财政体制改革的新形势，培育壮大县市区财源，着力发展中心城区财源，做大收入“蛋糕”，增强财政保障能力。依法加强财税征管，严格执行《关于进一步规范市直单位财政管理的意见》，进一步加强财政监督，严格项目资金管理。加快完善国有资产监管体系，规范产权管理，积极开展资本运作，确保国有资产保值增值。

　　（八）加快促进民生改善

　　进一步为民办好实事。严格对照省里下达的任务要求，抓好责任分解，加强资金配套，强化督促检查，确保民生项目全面落实。

　　进一步提高就业和社会保障水平。多渠道开发就业岗位，鼓励自主创业，完善公共就业和社会保障服务体系，重点帮助零就业家庭和就业困难群体就业，完善失地农民就业培训和社会保障政策，鼓励支持返乡农民工和大中专毕业生的就业创业，确保城镇新增就业3.3万人，新增农村劳动力转移就业7.2万人以上。进一步扩大养老、失业、医疗、工伤、生育和新型农村社会养老保险覆盖面，加快残疾人社会保障体系和综合服务体系建设，提高社会保障水平。建立健全离退休干部社区“四就近”服务管理。加强老年教育。关爱留守儿童。新增廉租住房租赁补贴1000户、廉租住房7259套，新建经济适用住房450套、公共租赁住房100套。扩大住房公积金覆盖面，发挥资金效益。

　　进一步发展各项社会事业。建立健全义务教育经费保障机制，逐步理顺教育管理体制，争取在城南新区、吉星路、娄底经济开发区分别新建一所高中、小学和民办学校，有效缓解中心城区大班额问题。全面完成医药卫生体制改革三年重点工作年度任务，完善新型农村合作医疗制度，大力推进城南医院建设。加强医德医风建设，健全农村医疗卫生、城市社区与公共卫生服务体系，做好传染病防控工作，提高突发公共卫生事件处置能力。全面加强人口和计划生育工作，稳定低生育水平，降低出生性别比，提高出生人口素质。做好湖南省第十二届运动会和第九届残疾人运动会的各项筹备工作。加快文化体制改革。推进市文化馆、图书馆、博物馆、艺术馆等重点文化设施建设。加大有线电视双向网改、城乡有线电视网络整合，提升广播影视公共服务传播能力。完善物价调节机制，确保物价指数涨幅控制在4%左右。大力发展慈善事业。深入推进殡葬改革。扎实推进食品药品安全专项整治，确保食品药品安全。重视革命老区工作。发展老龄事业。扎实推进第六次人口普查。做好外事侨务、民族、宗教、对台、地方志、红十字会、档案、保密、仲裁、地震等工作，重视和支持工会、共青团、妇联工作，大力发展妇女儿童事业。

　　进一步抓好安全生产。严格依法监管，严肃责任追究，全面推进“示范乡镇”、“示范县市”创建活动，夯实安全基础，加快“科技兴安”步伐，实现安全生产形势持续好转。落实安全生产责任，继续深化煤矿、道路交通、非煤矿山、烟花爆竹、危险化学品、特种设备、消防等重点行业领域安全专项整治，严防重特大事故发生。

　　进一步维护社会稳定。继续推进信访老案集中治理，建立健全维护群众权益和社会稳定风险评估机制，全面推行人民调解、司法调解、行政调解“三调联动”，从源头上预防和化解信访矛盾。健全完善重大突发事件预警机制和应急体系，提高预防和处置水平。完善社会治安防范体系，加强社会治安综合治理，推进社会管理创新，严厉打击“三强”，集中打击暴力恐怖、黑恶团伙、黄赌毒和多发性侵财犯罪，进一步提高人民群众的安全感。加强军队和国防后备力量建设，开展国防教育，增强全民国防观念。做好人民防空工作，提高城市防护能力。

四、加强政府自身建设

　　站在新的历史起点，机遇与挑战并存。必须按照科学、民主、依法行政的要求，努力建设服务政府、法治政府、责任政府和廉洁政府。

　　切实强化工作创新。倡导终身学习理念，鼓励大胆闯、大胆试、大胆干，着力突破资金、用地、人才、技术、环境等瓶颈制约。坚决破除“坐、等、靠、要”的意识，坚决破除小富即安、看摊守业的观念，坚决破除求稳怕乱、怕担责任的思想。牢固树立敢为人先、敢于担当的工作理念，以思想的大解放、观念的大更新、工作的大创新，促进全市的大发展。

　　切实强化服务保障。进一步简化审批流程，严格限时办结，推行项目前期工作代理制，完善“一厅式”办公、“一条龙”服务、“一票制”收费等制度和重大项目审批“绿色通道”，切实提高服务效率。大力推进政企分开、政资分开、政事分开、政府和市场中介组织分开，为各类市场主体创造公平竞争的环境。深入了解民情、倾听民意，及时解决企业和群众关心的热点难点问题。关心基层干部，支持基层工作，把更多的人力、财力、物力投向基层。

　　切实强化作风转变。下大力气精简会议，压缩文件。加强调查研究，做到知实情、谋实招、重实干、求实效。强化执行意识，确保政令畅通，对既定事项一抓到底、抓出成效。加强政府绩效评估，加大监察、督查力度，严格行政问责，落实目标责任。加强队伍建设，转变干部作风。坚持厉行节约，反对铺张浪费，真正把有限的资金、资源用到加快发展、改善民生上。

　　切实强化依法行政。全面启动“六五”普法工作，推进依法治市，建设法治娄底。规范执法行为，强化执法监督，做到依法决策、依法办事。认真落实市人大及其常委会各项决议决定，依法报告工作。自觉接受人大法律监督、工作监督和政协的民主监督。广泛听取和积极采纳各民主党派、工商联、无党派人士和各人民团体的意见，主动接受新闻舆论监督和社会公众监督，让政府权力在阳光下运行。严格落实党风廉政建设责任制，建设廉洁政府，加强审计监督，把全部政府性资金和资产纳入审计范围，健全反腐倡廉长效机制。

　　各位代表！过去的成就令人鼓舞；未来的目标催人奋进。我坚信，有市委的坚强领导，有市人大、市政协的监督支持，有全市人民的共同努力，我们一定能够圆满完成今年和“十二五”时期的各项目标任务，一定能够创造出无愧于时代、无愧于人民的新业绩，一定能够迎来娄底更加美好、灿烂的明天！
